# Effects of Dietary Fiber Supplementation on Gut Microbiota and Bowel Function in Healthy Adults: A Randomized Controlled Trial

**DOI:** 10.3390/microorganisms13092068

**Published:** 2025-09-05

**Authors:** Ryo Inoue, Kenta Suzuki, Masachika Takaoka, Michihiro Narumi, Yuji Naito

**Affiliations:** 1 Laboratory of Animal Science, Department of Applied Biological Sciences, Faculty of Agriculture, Setsunan University, Hirakata-shi 573-0101, Osaka, Japan; 2 Innovation Development Department, Mizkan Co., Ltd., Handa-shi 475-8585, Aichi, Japan; kenta_suzuki@mizkan.co.jp (K.S.); masachika_takaoka@mizkan.co.jp (M.T.); narumi@mizkan.co.jp (M.N.); 3 Department of Human Immunology and Nutrition Science, Kyoto Prefectural University of Medicine, Kyoto-shi 602-8566, Kyoto, Japan; ynaito@koto.kpu-m.ac.jp

**Keywords:** gut microbiota, bowel function, dietary fiber, randomized, double-blind, controlled trial

## Abstract

This randomized, double-blind, controlled trial evaluated the effects of 4-week dietary fiber supplementation on gut microbiota, bowel-related quality of life, and secondary outcomes, including sleep and skin condition. A total of 105 healthy adults received either low-fiber foods (2.2 g/day total fiber, 1.2 g/day fermentable fiber) or high-fiber foods (8.2 g/day total fiber, including 6.4 g/day fermentable fiber). Gut microbiota was analyzed by 16S rRNA sequencing. Outcomes included stool diary, JPAC-QOL (Japanese version of the Patient Assessment of Constipation Quality of Life), OSA-MA (Oguri-Shirakawa-Azumi sleep inventory MA version), skin questionnaires, and fecal organic acids. The high-fiber group showed significant improvements in JPAC-QOL and increases in SCFA-associated genera such as *Anaerostipes*, *Bifidobacterium*, and *Fusicatenibacter*. These taxa positively correlated with other beneficial bacteria, including *Faecalibacterium*, suggesting ecological cooperation. The effects on sleep and skin were limited but correlated with beneficial bacteria, implying possible gut–brain and gut–skin axes involvement. This study demonstrated that short-term fiber supplementation meaningfully improved the bowel-related quality of life and beneficially modulated the gut microbiota in healthy adults. Although the systemic effects were modest, microbial shifts suggest that higher fiber intake may provide broader health benefits with longer interventions. This study was registered in the UMIN Clinical Trial Registry (UMIN000054712).

## 1. Introduction

Dietary fiber is broadly defined as the indigestible portion of plant-derived food that escapes digestion in the upper gastrointestinal tract and reaches the colon, where it serves as the primary substrate for microbial fermentation [1]. It is an essential component of human nutrition, known not only for its role in maintaining gastrointestinal functions but also for its profound impact on the gut microbiota [2]. Dietary fibers can be categorized into fermentable fibers, which are utilized by gut bacteria as substrates for fermentation, and non-fermentable fibers, which contribute to stool bulking and normalizing intestinal transit without microbial fermentation. The typical examples for the former are inulin and pectin, while cellulose is a representative non-fermentable fiber [3].

The gut microbiota refers to the dense and diverse community of microorganisms, particularly bacteria, inhabiting the intestinal tract, most notably the colon. Over the past two decades, research has revealed that the gut microbiota plays a critical role in maintaining human health, but also may contribute to disease pathogenesis [4]. Indeed, dysbiosis, generally defined as an imbalance or disruption in the composition, function, or diversity of a microbial community, has been implicated not only in gastrointestinal disorders but also in systemic conditions such as type 2 diabetes, obesity, and autism spectrum disorder [4,5].

Accumulating evidence suggests that dietary fiber, particularly fermentable dietary fiber, modulates the composition of gut microbiota by stimulating the growth of beneficial gut microbes, particularly fiber-degrading bacteria. For example, inulin has been reported to increase the number of *Bifidobacterium* [6], while gum arabic stimulates the growth of lactobacilli in addition to *Bifidobacterium* [7]. The fermentation of dietary fiber by these bacteria leads to the production of short-chain fatty acids (SCFAs), including acetate, propionate, and butyrate [1]. These metabolites play crucial roles in supporting colonic epithelial integrity, regulating immune responses, and influencing the host metabolism [8].

In addition to its microbiota-modulating effects, dietary fiber is also known to influence bowel habits. It is reported that dietary fiber can alleviate not only constipation but also diarrhea [1]. Thus, dietary fiber is expected to contribute to gastrointestinal health through both microbial and mechanical pathways.

Although two major effects of dietary fiber, i.e., the effects on the gut microbiota and the bowel function, have been extensively studied [9,10,11], they have rarely been evaluated simultaneously [12,13,14]. Especially in healthy populations, integrative trials that assess both microbial and functional outcomes within a unified framework are still lacking. In particular, a few randomized controlled trials have concurrently assessed microbiota profiles and the bowel-related quality of life using validated instruments such as the Patient Assessment of Constipation Quality of Life (PAC-QOL) questionnaire.

Beyond gastrointestinal outcomes, dietary fiber is also increasingly studied for its potential impact on broader systemic health domains, including sleep quality and skin condition. These effects may be mediated via the gut-brain and gut-skin axes, both of which are influenced by microbial metabolites and host–microbe interactions [15,16,17]. However, the evidence for such effects by dietary fiber intervention in healthy individuals remains preliminary and largely exploratory.

To address these gaps, we conducted a randomized, controlled intervention trial using healthy adults to evaluate the effects of 4-week supplementation with dietary fiber, especially enriched by fermentable fiber. According to the Japan National Health and Nutrition Survey 2023 [18], the mean daily intake was 18.8 g in men and 16.9 g in women, with relatively large standard deviations (7.4 g and 6.5 g, respectively). Therefore, to ensure the average intake of dietary fiber exceeded the recommendation level (22 g for men, 18g for women), we designed the intervention to provide approximately 8 g/day of additional dietary fiber for the group having a fiber-enriched diet in this study. The primary objectives were to (1) assess whether fiber intake modulates the gut microbiota composition and (2) determine whether these changes correspond to improvements in the bowel-related quality of life. As secondary objectives, we explored potential effects on sleep quality and skin condition.

## 2. Materials and Methods

### 2.1. Ethics Statements and Participants

The present study was a double-blind, randomized, controlled trial conducted from 29 August 2024 to 26 September 2024. The sample size calculation for this study was based on the method using standardized effect sizes as presented in the DELTA2 guidance [19]. The cutoff point for the standardized effect size was set at Cohen’s d = 0.80, following Cohen’s recommendations. Assuming a significance level (α) of 5% and a statistical power (1-β) of 90%, the minimum required sample size was calculated to be 34 participants per group (68 in total). In this study, securing sufficient statistical power was prioritized. At the same time, considering budgetary and resource constraints, an efficient sample size design was adopted. As a result, the target sample size was set at 50 participants per group (100 in total), which was confirmed to provide a statistical power of 97.7%. Additionally, accounting for an estimated dropout rate and protocol deviations of approximately 10% during the study period, the final planned sample size was set at 55 participants per group (110 in total). This work was registered in the UMIN Clinical Trial Registry (UMIN000054712; registered on 26 August 2024) and approved by the ethical committee of Takara Clinic (Tokyo, Japan; Approval Number: 2405-06954-0018-11-TC), Mizkan Holdings Co., Ltd. (Approval Number: 24-E001), and Setsunan University (Approval Number: 2024-112).

Participants were recruited via an online website (https://www.go106.jp/, accessed on 1 September 2025) from 1 July to 29 July 2024. The inclusion criteria were healthy males or females aged between 20 and 50 years whose weekly defecation frequency was between 4 and 10 times. Exclusion criteria included (1) taking treatment for or having a history of malignant tumors, heart failure, or myocardial infarction, (2) implanted pacemakers or implantable cardioverter-defibrillators, (3) receiving treatment for chronic conditions such as arrhythmia, liver disorders, chronic kidney disease, cerebrovascular disorders, rheumatic diseases, diabetes mellitus, dyslipidemia, and hypertension, (4) consuming Foods for Specified Health Uses (FOSHU) or foods with functional claims, (5) taking medications (including traditional herbal medicines) or dietary supplements, particularly antibiotics or gastric acid suppressants, (6) having allergies to pharmaceuticals or foods related to the test diets such as wheat, eggs, milk, soybeans, chicken, pork, gelatin, seafood, buckwheat, yam, almonds, oranges, or peaches, (7) being pregnant, breastfeeding, or planning to become pregnant during the study period, (8) having participated in another clinical trial within 28 days prior to the date of consent, or those planning to participate in another trial during the study period, (9) having consumed dietary fiber-rich health foods (e.g., inulin, oligosaccharides, indigestible dextrin) within the past month, and (10) being deemed unsuitable for participation for any other reasons.

### 2.2. Randomization and Intervention

**Figure 1:**
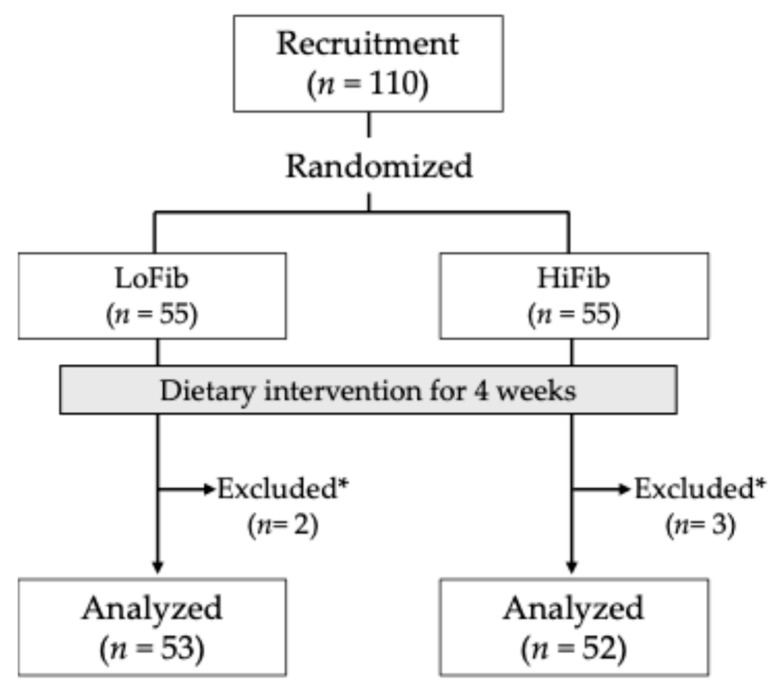
Flow chart of study participants.* Participants who did not provide samples or questionnaires due to personal reasons were excluded from downstream analyses.

The initial participants were randomly assigned into two groups: low-fiber group (LoFib, *n* = 55) and high-fiber group (HiFib, *n* = 55) (Figure 1) according to a randomization table generated by a computer. The randomization table was created using the R programming originally developed by a contract research organization (Ortho Medico Co., Ltd., Tokyo, Japan) based on a general block randomization method with variable block sizes. The allocation ratio between the two groups was 1:1. The correspondence between groups and blocks was securely retained by Ortho Medico and remained concealed from both participants and researchers until the completion of all analyses. The height and body weight of the participants were measured from 14 July to 3 August 2024 at Takara Clinic.

To avoid loss of appetite for the test diets, 5 different types of foods or drinks were used in this study (Table 1). The fiber fortification for test diets of the HiFib group was mainly based on inulin but also contained other dietary fibers such as resistant dextrin and isomalto-oligosaccharides (Fibee^®^; Mizkan, Handa, Japan). For the 4-week intervention, participants received a package containing seven test foods (two cereals, two teas, one cookie, one noodle, and one waffle) at the beginning of each week. They were asked to consume one item per day at their convenience, without specific instructions regarding timing or meal context. The HiFib group ingested 57.6 g of dietary fiber in the test diet per week (average 8.2 g/day; Fermentable fiber 6.4 g/day), while the LoFib group ingested 15.7 g per week of dietary fiber (average 2.2 g/day; Fermentable fiber 1.2 g/day).

The fiber content per test food for the HiFib group was determined based on the amount that could be realistically incorporated into products of a size commonly found on the market, while maintaining acceptable taste and palatability. In contrast, test foods for the LoFib group were prepared without fiber fortification, and their fiber content was derived only from the ingredients naturally present in the products. In this study, LoFib was regarded as the control.

According to the previous studies [20,21], the effects of dietary fiber on our primary outcomes, namely gut microbiota and bowel movements, have been reported to appear as early as 2–4 weeks of intervention. Therefore, the intervention period in this study was set for 4 weeks.

### 2.3. Questionnaires

Dietary intake was assessed by food frequency questionnaires a week before the start of the intervention. A 66-item Food Frequency Questionnaire (short-FFQ) [22] was used, and the nutrient intake of each participant was calculated with designated computer software (FFQ NEXT, Kenpakusha, Tokyo, Japan).

Subsequent questionnaires were completed by the participants at the beginning (week 0), the 2nd week (week 2), and the end (week 4) of the intervention. The questionnaires were (1) Stool diary (Table 2), (2) Japanese version of the Patient Assessment of Constipation Quality of Life (JPAC-QOL) [23], (3) Ogri-Shirakawa-Azumi sleep inventory MA version (OSA-MA) [24], and (4) Skin condition (Table S1).

### 2.4. Fecal Microbiota

#### 2.4.1. Measurement of Fecal Organic Acid Concentrations

Fecal samples were obtained using dedicated scoop-and-container kits (Sarstedt K.K., Tokyo, Japan) at weeks 0, 2, and 4 of the intervention. Throughout the handling process, samples were kept at 4 °C and brought to the laboratory within 24 h of collection. Organic acids concentration in feces, including acetate, propionate, iso-butyrate, butyrate, iso-valerate, valerate, succinate, lactate, and formate, was analyzed as per Miura et al. [25].

#### 2.4.2. Analysis of the Fecal Microbiota

Fecal microbial DNA was extracted with a Maxwell^®^ RSC Fecal Microbiome DNA Kit (Promega, Tokyo, Japan) according to the manufacturer’s instructions. The V3–V4 region of the 16S rRNA gene was amplified using the primer set 341F (5′-CCTACGGGNGGCWGCAG-3′) and 805R (5′-GACTACHVGGGTATCTAATCC-3′) with Tks Gflex DNA Polymerase (TaKaRa bio, Kusatsu, Japan). PCR amplification was performed using the following thermal cycling conditions: an initial denaturation at 95 °C for 3 min, followed by 25 cycles consisting of denaturation at 95 °C for 30 s, annealing at 55 °C for 30 s, and extension at 72 °C for 30 s, concluding with a final elongation step at 72 °C for 5 min. The resulting amplicons were purified using NucleoFast96 PCR plates (TaKaRa Bio), and a subsequent indexing PCR was carried out using unique dual-index primer sets compatible with MiSeq sequencing, following Illumina’s standard protocol (Illumina, San Diego, CA, USA). The amplicons after indexing PCR were purified and normalized using the SequalPrep Normalization Plate Kit (Life Technologies, Tokyo, Japan) and pooled at equimolar concentrations. The pooled library was further cleaned with AMPure XP magnetic beads (Beckman-Coulter, Brea, CA, USA). The resulting purified library was subjected to 285 bp paired-end sequencing on the Illumina MiSeq platform using the MiSeq Reagent Kit v3.

Data obtained from the MiSeq sequencing were analyzed as per Miura et al. [25] with some exceptions. In the present study, the version of QIIME2 [26] used was 2024.5, and the taxonomy of ASVs (amplicon sequence variants) was assigned against Greengenes2 [27].

### 2.5. Statistical Analysis

Scores for questionnaires, fecal organic acid concentrations, alpha-diversity indices of microbiota, and taxonomic distribution of bacteria based on relative abundances (genus level) between groups were compared with the Wilcoxon signed-rank test. The data in the respective groups were first compared with Friedman’s test, and when its *p*-value was less than 0.05, the Wilcoxon matched pairs signed-rank test was used between weeks 0 and 2, and between weeks 0 and 4. Due to approximately 2g of dietary fiber being included in LoFib diets, small effects on the parameters evaluated were expected in the study. Therefore, the effect size (r equivalent) was calculated, and *p* < 0.05 and |r| > 0.3 was considered statistically significant with a meaningful effect.

For the beta-diversity, both weighted and unweighted UniFrac distances between weeks 0 and 2, and between weeks 0 and 4 were calculated for the paired samples. These distances were then compared between the LoFib and HiFib groups with the Wilcoxon signed-rank test. For this comparison, a *p* value lower than 0.05 was considered statistically significant.

Spearman’s correlations among scores for questionnaires, concentrations of fecal organic acids, and relative abundances of major bacterial genera were evaluated with R (https://www.R-project.org/, accessed on 1 September 2025). Based on Benjamini-Hochberg adjusted *p* values (*p* < 0.1), a correlation network for three beneficial bacterial genera that increased in the HiFib group (genera *Anaerostipes*, *Bifidobacterium* 388775, and *Fusicatenibacter*) was constructed with Gephi Software ver 0.10.1 [28]. In the present study, bacterial genera showing mean relative abundances greater than 1% in at least three of six datasets (two groups; LoFib and HiFib × 3 time points; week 0, 2, and 4) were regarded as major bacterial genera. For an easy interpretation, in correlation analysis, scores for JPAC-QOL and Skin questionnaires were inverted (multiplied by −1), so that higher values indicated a better status.

## 3. Results

### 3.1. Characteristics of Participants

Five participants who did not provide fecal samples or questionnaires because of personal reasons were excluded (Figure 1). Thus, a total of 105 participants (LoFib, *n* = 53; HiFib, *n* = 52) were included in the analyses. Age, height, weight, body mass index, and basal intake of dietary fiber of participants were not significantly different between groups (Table 2). No harmful effects were reported by the participants.

### 3.2. Comparisons Between Groups

There were no between-group differences that met the predefined criteria (*p* < 0.05 and |r| > 0.3) for any questionnaire at any sampling time point (Table 3 and Table S2).

For bacterial taxonomy, the relative abundance of the genus *Bifidobacterium* 388775 was significantly and meaningfully higher in the HiFib group when compared with the LoFib group at both weeks 2 and 4.

At weeks 2 and 4, the relative abundance of *Bifidobacterium* 388775 in the LoFib group was 1.854 ± 2.110% and 2.473 ± 3.270%, respectively, whereas in the HiFib group it was 4.008 ± 4.206% and 4.863 ± 4.841%, respectively.

### 3.3. Changes During the Intervention in Each Group

#### 3.3.1. Stool Diary

Scores for “Number of bowel movements last week” and “Amount of stool per bowel movement” significantly increased from week 0 to both weeks 2 and 4, regardless of the experimental group (Table 3). Scores for “Number of days having bowel movement last week” significantly increased from week 0 to week 4 in both groups (Table 3).

#### 3.3.2. JPAC-QOL

All scales of JAPC-QOL significantly decreased at both weeks 2 and 4 in comparison with week 0, regardless of the experimental group (Table 3). Regarding the subscale (questions), scores for 11 of 28 subscales (Q5–6, Q11, Q15, Q17, Q22–23) significantly decreased only in the HiFib group both at weeks 2 and 4 in comparison with week 0. Indeed, the effect size of all scales, especially the “Overall” scale, was higher in the HiFib group than in the LoFib group. Scores for Q1 decreased significantly only in the HiFib group from week 0 to week 4, while those for Q13 and Q24 decreased significantly in the LoFib group from week 0 to week 2 and from week 0 to week 4, respectively.

#### 3.3.3. OSA-MA

No changes were found in either the major scales or subscales of OSA-MA regardless of the experimental groups (Table 3).

#### 3.3.4. Skin Condition

In the LoFib group, scores for “Skin dryness”, “Skin clarity”, “Skin smoothness”, and “Makeup adherence” significantly decreased (improved) at week 2 (Table 3). At week 4, scores for “Skin dryness” significantly decreased. Although the effect size was slightly less than 0.3, scores for “Noticeable pores” and “Noticeable spots” decreased with the *p*-value lower than 0.05 at week 2 and week 4, respectively.

In the HiFib group, scores for “Skin clarity” significantly decreased both at weeks 2 and 4 (Table 3). Scores for “Noticeable pores” and “Facial swelling” at week 2 and the score for “Skin smoothness” at week 4 decreased with the *p*-value lower than 0.05, but the effect size was slightly lower than 0.3.

#### 3.3.5. Effect of the Intervention on Fecal Organic Acids

At week 2, butyrate, iso-butyrate, and iso-valerate concentrations significantly increased in the HiFib group (Table 3). No significant differences were found in the LoFib group both at weeks 2 and 4, and in the HiFib group at week 4.

#### 3.3.6. Effect of the Intervention on Fecal Microbiota

No significant changes were found in the indices for alpha-diversity (Chao1 and Shannon) during the intervention period in both the LoFib and HiFib groups.

**Figure 2:**
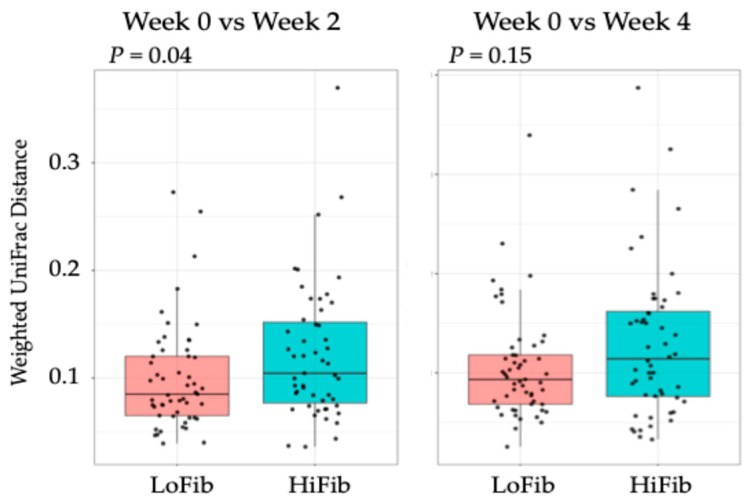
Weighted UniFrac distance between weeks 0 and 2, and between weeks 0 and 4 in the paired samples.

Regarding beta-diversity, the distance based on weighted UniFrac metrics between weeks 0 and 2 in paired samples was significantly higher in the HiFib group than in the LoFib group, meaning the changes in the bacterial composition were greater in the HiFib group than in the LoFib group (Figure 2). In both groups, distances based on unweighted UniFrac did not differ between weeks 0 and 2 and between weeks 0 vs. 4.

At week 4, the distance from week 0 in paired samples was still higher in the HiFib group than in the LoFib group, but the differences between groups were not statistically significant (Figure 2).

The number of bacterial genera whose relative abundances changed during the intervention period was higher in the HiFib group than in the LoFib group (Table 4). In the LoFib group, the relative abundances of 4 and 3 genera significantly changed at weeks 2 and 4, respectively, in comparison with week 0. For example, the relative abundance of genus *Anaerobutyricum* decreased at week 2 but increased at week 4. Genus *Blautia* A 141781 significantly increased at week 4 when compared with week 0.

In the HiFib group, the relative abundances of 10 and 13 genera changed at weeks 2 and 4, in comparison with week 0. The relative abundance of genus *Bifidobacterium* 388775 significantly increased at both weeks 2 and 4. In addition, the relative abundances of the genera *Anaerostipes* and *Fusicatenibacter* also significantly increased from week 0 to 4. Lastly, the dynamics of the genera *Anaerobutyricum* and *Blautia* A 141781 in the HiFib group were similar to those in the LoFib group.

### 3.4. Correlation Network

**Figure 3:**
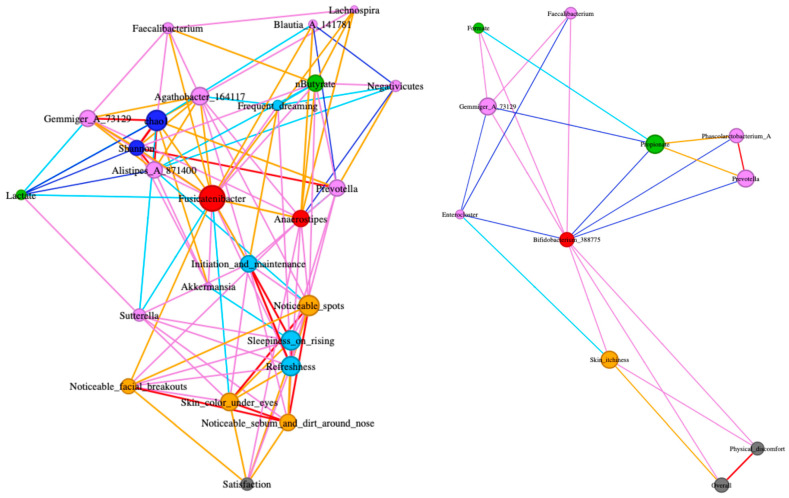
Correlation network among scores for questionnaires, concentrations of fecal organic acids, and relative abundances of major bacterial genera. Left panel: Network directly connected with genus Bifidobacterium 388775 extracted from the full network (see Figure S1). Right Panel: Network directly connected with either genus Anaerostipes or Fusicatenibacter extracted from the full network (see Figure S1). Node colors: Red: Anaerostipes, Bifidobacterium 388775, and Fusicatenibacter, Pink: Major bacterial genera except for the former three genera, Blue: Alpha diversity indices, Light green: Organic acids, Grey: JPAC-QOL, Cyan: OSA-MA, Orange: Skin condition. Scores for the stool diary were not correlated with three genera. Edge colors indicate Spearman’s correlation coefficient: Red: >0.4, Pink: 0.4–0.2, Orange: 0–0.2, Cyan: −0.2–0, Blue: −0.4–−0.2.

The abundances of genera *Anaeropstipes*, *Bifidobacterium* 388775, and *Fusicatenibacter* positively correlated with well-known beneficial bacteria, such as genera *Faecalibacterium* and *Prevotella*. Genera *Anaerostipes* and *Fusicatenibacter* positively correlated with indices for alpha diversity (Chao1 and Shannon) and the concentration of *n*-butyrate (Figure 3). These two genera also showed positive correlations with scores for some scales of OSA-MA and Skin questionnaires, indicating increased abundances of these genera related to a better status of sleep and some skin conditions. In contrast, the abundance of genus *Bifidobacterium* 388775 positively correlated with scores for two scales of JPAC-QOL, namely Overall and Physical discomfort.

## 4. Discussion

The present randomized, controlled trial evaluated the effects of 4-week dietary fiber supplementation on the gut microbiota composition and the bowel-related quality of life in healthy adults.

Importantly, our study is among the few to simultaneously assess both the microbiota and functional bowel outcomes within the same healthy population. The findings indicated two major effects of dietary fiber: modulation of the gut microbiota and improvement of the subjective bowel function, potentially mediated through interrelated mechanisms.

Consistent with previous reports [11,29], we observed a positive modulation of fiber-degrading and SCFA-producing bacterial genera, including *Bifidobacterium* 388775, *Anaerostipes*, and *Fusicatenibacter*, alongside improvements in JPAC-QOL scores. Notably, beneficial changes were observed in both the LoFib and HiFib groups, although the magnitude was clearly higher in the HiFib group.

The HiFib group, which achieved an average fiber intake of 8.2 g/day (including 6.2 g/day of fermentable fiber) in addition to the habitual dietary fiber, demonstrated stronger effects on both microbial diversity and JPAC-QOL subscales. Notably, *Bifidobacterium* 388775, a well-known inulin utilizer [30], showed a sustained increase during the experimental period, correlating positively with improvements in the “Overall” and “Physical discomfort” scales of the JPAC-QOL. This fact supports the role of *Bifidobacterium* as a key mediator of fiber’s effects on defecation quality.

An interesting finding was the increase in the genera *Anaerostipes* and *Fusicatenibacter* in the HiFib group. *Fusicatenibacter* can degrade various saccharides and produce lactate, acetate, and succinate [31], while *Anaerostipes* can produce butyrate from lactate and acetate [32]. Succinate can be utilized by other gut bacteria and converted into propionate and butyrate [33]. Increases in fecal butyrate concentrations observed in the HiFib group at week 2 further support this functional shift, even if changes were not sustained at week 4. This pattern suggested that *Anaerostipes* and *Fusicatenibacter* could be potential contributors to SCFA pools during fiber supplementation, and that butyrate production may partially explain the observed improvements in the bowel-related quality of life.

Moreover, correlation analysis revealed that *Anaerostipes*, *Bifidobacterium* 388775, and *Fusicatenibacter* were positively associated with other beneficial genera such as *Faecalibacterium* and *Prevotella* [34,35]. These inter-genus associations suggest a possible ecological cooperation within the gut microbiome, where cross-feeding and syntrophic relationships stabilize community structures under higher fiber intake. In addition to *Fucicatenibacter*, *Bifidobacterium* is known to initiate butyrogenic microbial networks by degrading fermentable dietary fibers (e.g., inulins), thereby providing substrates for *Anaerostipes* and *Faecalibacterium*; the latter being a well-known butyrate producer [36]. Similarly, *Prevotella* species can break down complex plant polysaccharides into oligosaccharides, providing substrate for *Anaerostipes* and other butyrate-producing bacteria [35]. These observations reinforce the notion that dietary fiber does not act in isolation, but rather reshapes the cooperative microbiota ecosystem that supports gut homeostasis.

The transitions of enterotypes also support the above-mentioned notion. Although not a primary endpoint, we further evaluated the changes in the microbial community structure through Japanese enterotype transitions during the intervention (Figure S2) using a support vector machine–based classification [37]. A change in enterotypes was observed in 7 participants (13%) in the LoFib group at week 2, while about three times more participants (20 subjects; 39%) shifted enterotypes in the HiFib group. At week 4, the number of participants whose enterotypes changed was still higher in the HiFib group than in the LoFib group (LoFib 11 subjects vs. HiFib 23 subjects).

When exploratory subgroup analyses according to habitual fiber intake were performed, some interesting aspects were suggested. In participants with low habitual dietary fiber intakes (defined as <80% of the recommended level in the Dietary Reference Intakes for Japanese [38]), even the LoFib group showed beneficial effects on the gut microbiota and JPAC-QOL. This finding suggests that individuals with suboptimal habitual fiber consumption may respond to relatively small increases in fiber intake. In contrast, among participants with a higher habitual fiber intake (≥80% of the recommended level), only the HiFib group, which achieved a mean intake close to 25 g/day (near the European recommendation [39]) showed significant additional benefits, including an improvement in sleep length. Given that microbiota-derived SCFAs and other metabolites have been suggested to be involved in the gut–brain axis pathways [40], a more robust fiber dose might be needed to elicit systemic effects beyond compositional changes in the gut microbiota. Details regarding this subgroup analysis are provided in the Supplementary Text S1.

In terms of skin and sleep parameters overall, no clear benefits were detected except in a few questionnaire items. Yet correlation patterns suggested potential for improvement over a longer intervention: *Anaerostipes* and *Fusicatenibacter* were positively associated with some OSA-MA and skin condition scales, implying that maintenance of higher abundances in these taxa might benefit systemic health beyond the gut.

In addition, a correlation matrix shown in Figure S5 illustrates the interrelationships among the bowel-related quality of life, sleep parameters, skin condition, and stool diary measures. Consistent with the primary findings, JPAC-QOL scales showed strong positive associations with objective stool diary parameters, reinforcing the link between subjective and objective assessments of bowel function. Interestingly, several sleep quality indicators (particularly “sleep length” and “refreshing”) demonstrated positive correlations with certain scales for skin condition and JPAC-QOL, suggesting a potential gut–brain–skin axis connection [41]. While these cross-sectional associations do not prove causality, they support the concept that an improved gut function through dietary fiber intake could influence systemic health markers such as sleep and skin, potentially via shared immunological or metabolic pathways. These observations warrant further investigation, ideally in longer-term trials where such systemic effects may become more pronounced.

There were several limitations that warrant mention. First, no placebo group was set due to the dietary fiber naturally present in the ingredients of the test diets. While the LoFib group was designed to be the control group, its 2.2 g/day fiber content in the test diet was not negligible. Second, the 4-week duration of the intervention might have been insufficient to see durable systemic changes beyond the bowel function. Third, while 16S rRNA sequencing (used in the present work) achieves genus-level resolution, it cannot elucidate species- and strain-level or functional gene differences. Future studies using metagenomic or metabolomic approaches could help identify which bacterial species and functional pathways are most responsible for the observed health benefits.

## 5. Conclusions

Our results demonstrated that dietary fiber supplementation to healthy adults can meaningfully shift the gut microbiota structure, particularly increasing SCFA-producing genera, and improve the bowel-related quality of life within 4 weeks. The concurrent observation of these two effects, i.e., microbial modulation and bowel function improvement in individuals without overt gastrointestinal symptoms, is particularly noteworthy.

These effects were enhanced by higher fiber doses approaching European recommendations. Moreover, correlations among *Bifidobacterium*, *Anaerostipes*, *Fusicatenibacter*, and other beneficial genera, such as *Faecalibacterium* and *Prevotella*, highlight a potentially cooperative microbiota network supporting gut health. Although systemic outcomes like sleep and skin showed limited changes in the short timeframe, the microbial shifts we observed suggested that a longer intervention could potentially yield broader benefits via gut–brain and gut–skin pathways.

## Figures and Tables

**Table 1:** Energy and macronutrient composition of placebo and treatment diets.

	Cereal		Noodle		Cookie		Waffle		Tea	
	LoFib	HiFib	LoFib	HiFib	LoFib	HiFib	LoFib	HiFib	LoFib	HiFib
Energy, kcal	169.1	168.0	307.7	309.0	154.5	152.0	194.0	203.0	0.0	25.0
Protein, g	3.2	2.9	13.3	13.0	2.1	2.8	3.5	5.4	0.0	0.0
Fat, g	6.2	5.7	5.4	5.4	7.3	6.9	3.9	9.0	0.0	0.0
Carbohydrate, g	28.8	30.0	55.3	56.0	20.1	23.6	28.8	30.3	0.0	10.1
Dietary Fiber, g	3.8	7.3	5.3	8.7	1.1	7.5	1.7	10.6	0.0	8.1
Salt, mg	98.4	90.0	5700.0	5700.0	60.0	25.0	300.0	290.0	20.0	190.0
Source of Major Dietary Fiber	Oats Barley	Oats Barley Inulin	WF * Oat flour Soy flour	WF * Oat flour Inulin Soy flour		Inulin RD * OP *		IMO * OP * WF * PHP * CF *		Inulin RD *

* WF: Whole wheat flour, RD: Resistant dextrin, OP: Okara powder, IMO: Isomalto-oligosaccharides, PHP: Psyllium husk powder, CF: Chickpea flour.

**Table 2:** Baseline information of participants.

Item	LoFib		HiFib		*p* Value *	
	Male	Female	Male	Female	Male	Female
N (Male/Female)	20	33	17	35	N/A	N/A
Age	38.5 ± 8.5	42.0 ± 7.4	39.0 ± 6.8	42.8 ± 6.5	0.91	0.96
Height, cm	173.9 ± 5.0	159.7 ± 5.8	172.3 ± 5.0	158.1 ± 6.3	0.40	0.32
Body weight, kg	71.0 ± 12.2	53.8 ± 8.5	69.3 ± 7.1	54.5 ± 10.0	0.85	0.31
Body mass index	23.5 ± 4.3	21.1 ± 3.3	23.3 ± 1.9	21.7 ± 3.3	0.84	0.17
Basal intake of dietary fiber, g	13.2 ± 1.7	14.1 ± 1.2	13.9 ± 2.2	14.5 ± 2.0	0.34	0.63

* *p* values were calculated with the Wilcoxon signed-rank test.

**Table 3:** Data from questionnaires and the analysis of organic acid concentrations at weeks 0, 2, and 4.

		LoFib										HiFib									
		Week 0		Week 2		Week 4		Week 0 vs. 2		Week 0 vs. 4		Week 0		Week 2		Week 4		Week 0 vs. 2		Week 0 vs. 4	
								*p* Value	Effect Size	*p* Value	Effect Size							*p* Value	Effect Size	*p* Value	Effect Size
Stool Diary	Number of days having bowel movement last week	5	(5–7)	6	(5–7)	7	(5–7)	0.24	0.16	0.03	0.30	5	(4–7)	6	(5–7)	6	(5–7)	0.06	0.26	0.01	0.37
	Number of defecation times last week	6	(5–8)	7	(6–9)	8	(6–9)	<0.01	0.43	<0.01	0.40	6	(5–7)	7	(5–8)	7	(5–10)	0.03	0.29	<0.01	0.39
	Amount of stool per bowel movement	19	(12–31)	28	(19–44)	30	(17–51)	<0.01	0.47	<0.01	0.44	19	(14–28.5)	25	(16.75–39.75)	30.5	(20–43.25)	<0.01	0.51	<0.01	0.62
	Form of stool	5	(5–6)	5	(4–5)	5	(5–5)	0.90	0.02	0.30	0.14	5	(4–6)	5	(4–5)	5	(4.75–5)	0.40	0.12	0.78	0.04
	Smell	3	(3–3)	3	(3–3)	3	(3–3)	0.30	0.14	0.48	0.10	3	(3–3)	3	(3–3)	3	(3–3)	0.18	0.19	0.18	0.18
	Feeling after defecation	2	(2–2)	2	(2–2)	2	(2–2)	0.80	0.03	0.13	0.21	2	(2–2)	2	(1–2)	2	(2–2)	0.05	0.27	0.36	0.13
JPAC-QOL	Overall	22	(15–37)	20	(13–28)	18	(12–26)	<0.01	0.46	<0.01	0.57	29.5	(19.75–43.75)	24.5	(14.5–34)	20	(11.5–30.5)	<0.01	0.73	<0.01	0.72
	Physical discomfort	2	(1–4)	1	(0–3)	1	(0–2)	0.01	0.38	<0.01	0.49	3	(1–6)	2	(1–3.25)	2	(0–3)	<0.01	0.50	<0.01	0.60
	Psychosocial discomfort	1	(0–4)	1	(0–4)	1	(0–5)	0.03	0.30	0.01	0.35	2	(1–9)	1.5	(0.75–6)	1	(0–5)	<0.01	0.58	<0.01	0.57
	Worries concerns	6	(4–11)	4	(4–9)	4	(3–8)	<0.01	0.43	<0.01	0.45	10	(4–17)	7	(4–11.25)	5.5	(3–11)	<0.01	0.48	<0.01	0.55
	Satisfaction	12	(9–15)	10	(8–14)	10	(6–13)	0.03	0.31	<0.01	0.45	13	(10.75–16)	13	(8–14)	10.5	(6.75–14)	<0.01	0.44	<0.01	0.57
OSA-MA	Sleepiness on rising	17.1	(13.6–20.4)	17.3	(13.9–20.6)	17.8	(14.6–20.3)	0.55	0.08	0.15	0.20	16.15	(13.55–20.5)	17.5	(13.58–20.47)	17.1	(13.8–20.22)	0.50	0.09	0.99	0.00
	Initiation and maintenance	18.9	(13.7–20.5)	18	(15.9–21.3)	18.3	(14.7–21.9)	0.65	0.06	0.33	0.13	16.9	(14.83–19.8)	17.45	(13.8–19.62)	15.85	(12.75–20.65)	0.65	0.06	0.50	0.09
	Frequent dreaming	25.5	(20.8–29.5)	25.5	(18.8–29.5)	27.5	(18.8–29.5)	0.38	0.12	0.65	0.06	23	(18.78–29.5)	22.8	(18.45–29.5)	23.5	(16.88–29.5)	0.72	0.05	0.60	0.07
	Refreshness	16.4	(13.2–19.7)	16.2	(13.2–19.8)	17.2	(14.4–20.1)	0.95	0.01	0.06	0.26	15.7	(13–18.4)	15.55	(12.52–18.95)	15.2	(12–19.33)	0.70	0.05	0.85	0.03
	Sleep length	17.8	(16–21.5)	19.7	(16–21.5)	18.2	(16–21.5)	0.98	0.00	0.62	0.07	16	(14.2–20.6)	17.8	(14.2–21.35)	17.8	(16–20.38)	0.91	0.02	0.54	0.09
Skin questionnaire	Q1. Overall skin condition	4	(1–6)	3	(1–5)	3	(2–5)	0.82	0.03	0.65	0.06	3	(1.75–6)	4	(2–5.25)	5	(3–6)	0.51	0.09	0.08	0.24
	Q2. Skin roughness	4	(1–6)	4	(1–5)	4	(2–6)	0.27	0.15	0.88	0.02	3	(2–6)	3	(2–6)	3.5	(2–6)	0.84	0.03	0.84	0.03
	Q3. Skin itchiness	2	(0–3)	2	(0–4)	2	(0–4)	0.39	0.12	0.46	0.10	2	(0–4.25)	2.5	(0–5)	2	(0–4.25)	0.18	0.19	0.32	0.14
	Q4. Noticeable wrinkles	5	(2–7)	3	(1–6)	4	(2–6)	0.15	0.20	0.10	0.23	5	(3–7)	4	(2–7)	4.5	(3–6)	0.14	0.21	0.23	0.17
	Q5. Noticeable spots	6	(3–8)	6	(2–7)	5	(3–7)	0.29	0.14	0.04	0.29	6	(2.75–7.25)	5	(3–6.25)	4	(3–6.25)	0.21	0.17	0.21	0.17
	Q6. Noticeable facial breakouts	3	(1–6)	3	(0–6)	3	(1–6)	0.16	0.19	0.28	0.15	5	(1–7)	3	(1.75–6)	4	(1.75–6)	0.44	0.11	0.97	0.01
	Q7. Noticeable sebum and dirt around nose	6	(3–7)	5	(3–7)	5	(3–6)	0.06	0.26	0.09	0.23	6	(4–8)	5	(3–7)	5	(3–7)	0.22	0.17	0.12	0.22
	Q8. Noticeable pores	6	(4–8)	5	(4–7)	5	(3–7)	0.04	0.28	0.09	0.24	6.5	(3.75–8)	5	(3–7)	5	(3–7)	0.04	0.28	0.12	0.22
	Q9. Skin dryness	4	(3–6)	3	(2–5)	3	(1–6)	0.01	0.37	0.01	0.33	4	(2–6)	3.5	(2–5)	4	(2–5)	0.66	0.06	0.64	0.06
	Q10. Facial swelling	4	(2–6)	3	(1–6)	3	(1–6)	0.18	0.18	0.40	0.12	5.5	(1.75–7)	4	(2–5)	4	(2–6)	0.04	0.28	0.13	0.21
	Q11. Skin color under eyes	5	(2–8)	5	(2–7)	5	(2–7)	0.39	0.12	0.10	0.22	5	(4–8)	5	(2–7)	5	(3–7)	0.05	0.27	0.07	0.25
	Q12. Skin firmness and elasticity	5	(5–7)	5	(4–6)	5	(3–7)	0.07	0.25	0.10	0.23	5	(4–7)	5	(3.75–7)	5	(3.75–6.25)	0.65	0.06	0.29	0.15
	Q13. Skin clarity	6	(5–8)	5	(4–7)	5	(3–8)	0.00	0.41	0.14	0.20	6	(5–8)	5	(4–7)	5	(4–7)	0.01	0.34	<0.01	0.45
	Q14. Skin smoothness	5	(4–7)	5	(3–6)	5	(3–6)	0.01	0.34	0.11	0.22	5	(4–6.25)	5	(4–6)	4	(3–6)	0.74	0.05	0.03	0.29
	Q15. Makeup adherence	5	(5–7)	5	(3–6)	5	(3–6)	0.00	0.41	0.10	0.23	5	(4–7)	5	(4–6)	5	(4–5)	0.32	0.14	0.28	0.15
Organic Acids	Succinate	1.45	3.16	2.23	6.25	1.73	6.05	0.89	0.02	0.87	0.02	1.40	3.07	0.92	1.68	2.02	5.03	0.39	0.12	0.98	0.00
	Lactate	0.26	0.65	0.11	0.21	0.26	1.32	0.32	0.14	0.41	0.11	0.26	0.84	0.11	0.20	0.33	1.83	0.25	0.16	0.07	0.25
	Formate	0.34	0.58	0.18	0.11	0.27	0.48	0.89	0.02	0.74	0.05	0.21	0.33	0.16	0.15	0.23	0.42	0.57	0.08	0.68	0.06
	Acetate	49.78	24.96	51.54	21.95	47.16	20.32	0.38	0.12	0.47	0.10	51.78	23.53	55.83	25.45	52.35	22.97	0.53	0.09	0.90	0.02
	Propionate	17.32	10.05	18.32	9.20	17.01	9.70	0.30	0.14	0.91	0.02	18.24	11.11	19.19	8.21	18.12	7.93	0.15	0.20	0.64	0.06
	isoButyrate	1.78	1.83	1.65	0.98	1.75	1.03	0.56	0.08	0.37	0.12	1.61	1.14	2.01	0.99	1.76	1.28	<0.01	0.49	0.51	0.09
	nButyrate	10.49	8.75	10.17	6.55	8.81	4.90	0.88	0.02	0.39	0.12	9.11	7.96	10.79	6.65	9.44	6.21	0.01	0.35	0.52	0.09
	isoValerate	1.66	1.41	1.63	1.23	1.81	1.23	0.89	0.02	0.35	0.13	1.70	1.40	2.08	1.27	1.73	1.50	<0.01	0.42	0.95	0.01
	nValerate	1.33	1.40	1.48	1.31	1.46	1.36	0.10	0.22	0.16	0.19	1.43	1.53	1.48	1.37	1.37	1.44	0.48	0.10	0.80	0.04
	Total SCFA	82.35	40.06	84.79	33.05	78.00	32.30	0.45	0.10	0.61	0.07	83.88	38.55	91.38	37.27	84.78	35.22	0.18	0.19	0.86	0.02

Values are expressed as median and interquartile range (IQR) except for organic acids (means and standard deviations). Values with grey backgrounds differ significantly and meaningfully compared with week 0 (*p* value < 0.05 and |Effect size| > 0.3). Scores for questionnaires and fecal organic acid concentrations in the respective groups were first compared with Friedman’s test, and when its *p*-value was less than 0.05, the Wilcoxon matched pairs signed-rank test was used between weeks 0 and 2, and between weeks 0 and 4. Only the results of the Wilcoxon matched pairs signed-rank test are shown.

**Table 4:** Bacterial genera whose abundance significantly changed during the intervention.

Taxon	Week 0	Week 2	Week4	Week 0 vs. 2		Week 0 vs. 4		
				*p* Value	Effect Size	*p* Value	Effect Size	
**LoFib group**								
*Anaerobutyricum*	0.468 ± 0.471	0.401 ± 0.520	0.729 ± 0.795	<0.01	0.380	<0.01	0.590	
*Blautia_A_141781*	5.280 ± 3.391	4.547 ± 3.309	7.136 ± 6.000	0.088	0.235	0.020	0.320	
*Enterocloster*	1.815 ± 1.675	1.690 ± 1.726	1.458 ± 1.512	0.463	0.101	0.017	0.328	
*Bacteroidaceae genus unclassified*	0.004 ± 0.026	0.007 ± 0.041	0.005 ± 0.032	0.018	0.325	0.361	0.125	
*Hungatella_A_128155*	0.169 ± 0.726	0.032 ± 0.099	0.060 ± 0.249	0.024	0.310	0.175	0.186	
*Ventrisoma*	0.015 ± 0.038	0.006 ± 0.023	0.008 ± 0.030	0.025	0.308	0.100	0.226	
**HiFib group**								
*Agathobaculum*	0.309 ± 0.309	0.291 ± 0.263	0.403 ± 0.305	0.392	0.120	<0.01	0.391	
*Anaerobutyricum*	0.424 ± 0.406	0.293 ± 0.306	0.746 ± 0.635	<0.01	0.400	<0.01	0.610	
*Anaerostipes*	0.673 ± 0.838	0.626 ± 0.652	1.141 ± 1.658	0.956	0.008	<0.01	0.475	
*Bariatricus*	0.147 ± 0.246	0.110 ± 0.184	0.192 ± 0.315	<0.01	0.363	0.082	0.243	
*Bifidobacterium_388775*	3.177 ± 3.453	4.008 ± 4.206	4.863 ± 4.841	<0.01	0.361	<0.01	0.389	
*Blautia_A_141781*	4.457 ± 3.151	3.559 ± 3.027	5.483 ± 3.105	<0.01	0.367	<0.01	0.381	
*Butyricimonas*	0.072 ± 0.147	0.099 ± 0.159	0.123 ± 0.312	<0.01	0.354	0.089	0.238	
CAG-41	0.079 ± 0.088	0.142 ± 0.187	0.127 ± 0.164	<0.01	0.342	0.073	0.251	
*Dorea_A*	0.461 ± 0.421	0.439 ± 0.459	0.543 ± 0.527	0.654	0.063	<0.01	0.346	
*Eubacterium_I*	0.057 ± 0.091	0.040 ± 0.072	0.072 ± 0.104	0.029	0.306	0.128	0.213	
*Fusicatenibacter*	1.267 ± 1.296	1.155 ± 1.403	1.593 ± 1.610	0.476	0.100	0.023	0.319	
*Enterobacteriaceae_A genus unclassified*	0.452 ± 1.575	0.098 ± 0.263	0.126 ± 0.688	0.094	0.235	0.028	0.308	
*Peptostreptococcaceae_256921 genus unclassified*	0.071 ± 0.113	0.082 ± 0.196	0.465 ± 2.113	0.758	0.043	0.023	0.318	
*Limivivens*	0.008 ± 0.026	0.014 ± 0.031	0.018 ± 0.039	0.196	0.181	<0.01	0.368	
*Oliverpabstia*	0.181 ± 0.444	0.089 ± 0.191	0.132 ± 0.300	0.029	0.305	0.344	0.133	
*Parasutterella*	0.830 ± 1.728	1.427 ± 2.875	1.244 ± 2.259	<0.01	0.475	0.051	0.274	
RUG115	0.008 ± 0.033	0.012 ± 0.071	0.042 ± 0.202	1.000	0.000	0.021	0.324	
*Ruminococcus_C_58660*	0.086 ± 0.240	0.106 ± 0.267	0.192 ± 0.465	0.666	0.060	0.031	0.302	
UMGS1375	0.105 ± 0.308	0.049 ± 0.141	0.053 ± 0.148	<0.01	0.385	0.019	0.328	

Bacterial genera of which relative abundances showed meaningful changes during the intervention are listed. Differences at the level of *p*-value < 0.05 and effect size > 0.3 were regarded as meaningful (grey background). Bold and underlined labels in Taxon column represent the intervention groups.

## Data Availability

Raw sequences have been deposited in the NCBI Sequence Read Archive under the BioProject ID PRJNA1300638 (available from 1 October 2025).

## Supplementary Materials

The following supporting information can be downloaded at: https://www.mdpi.com/article/10.3390/microorganisms13092068/s1. Table S1: Questionnaires for stool diary and skin condition. Table S2: Results of statistical analyses comparing groups based on questionnaire data and organic acid concentrations. Table S3: Data from questionnaires and the analysis of organic acid concentrations at weeks 0, 2, and 4 in four subgroups. Table S4: Bacterial genera in four subgroups, whose abundances significantly changed during the intervention. Figure S1: Correlation network among scores for questionnaires, concentrations of fecal organic acids, and relative abundances of major bacterial genera. Figure S2: Transitions of Japanese enterotype during the intervention. Figure S3: Flow chart of study participants including subgroup analyses. Figure S4: Weighted UniFrac distance between weeks 0 and 2, and between weeks 0 and 4 in the paired samples of four subgroups. Figure S5: Correlation matrix illustrating interrelationships among stool diary measures, bowel-related quality of life, sleep parameters, and skin condition scores. Text S1: Method and Results for Subgroup Analyses.
