# Supplementary material for: Effects of Dietary Fiber Supplementation on Gut Microbiota and Bowel Function in Healthy Adults: A Randomized Controlled Trial
Source: Microorganisms. 2025 Sep 5;13(9):2068. doi: 10.3390/microorganisms13092068 (PMC12471990; doi:10.3390/microorganisms13092068)
Supplement: Supplementary file 1 [file microorganisms-13-02068-s001.zip › Supplementary Figs.pptx]

## Slide 1
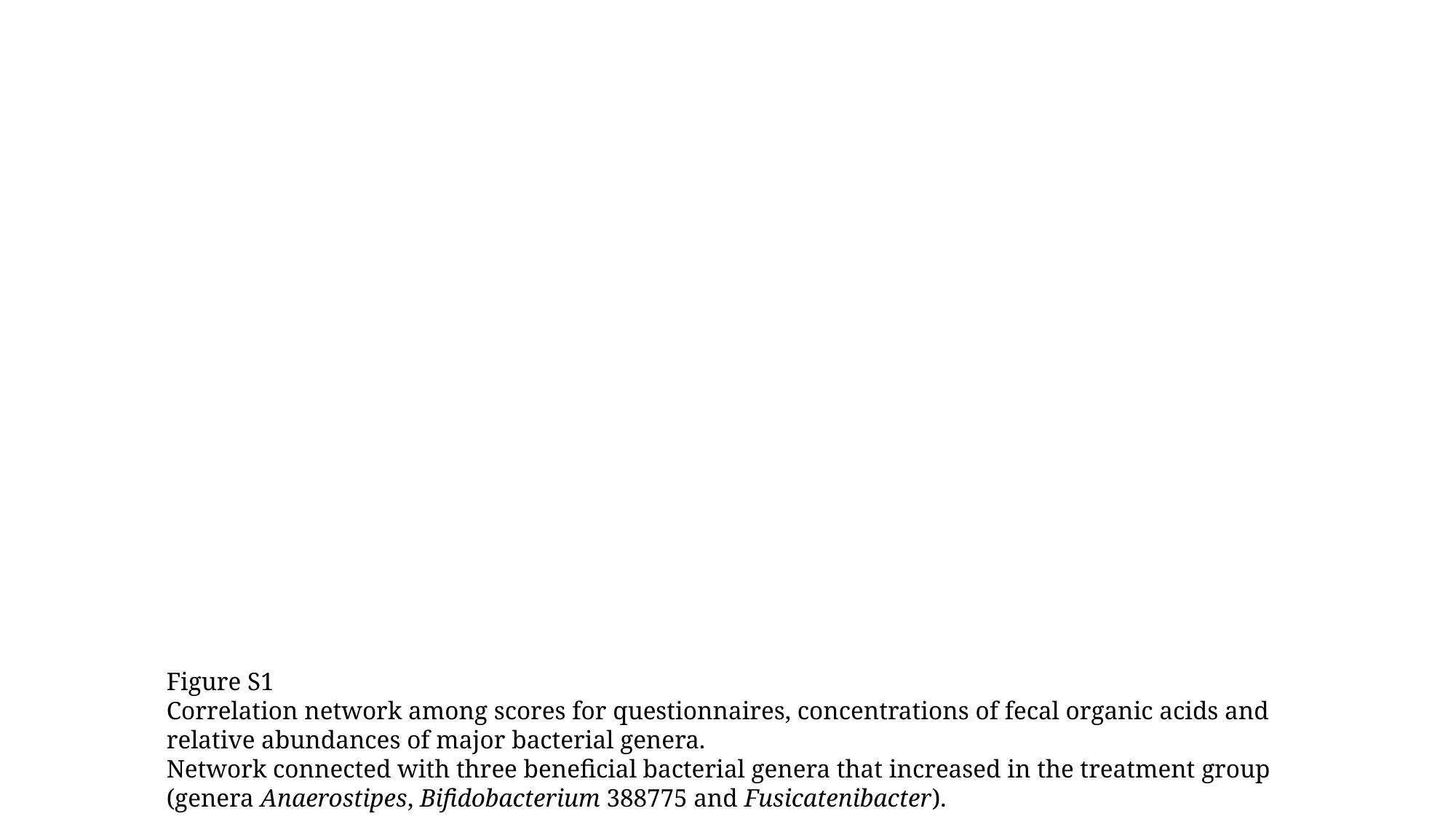

Figure S1
Correlation network among scores for questionnaires, concentrations of fecal organic acids and relative abundances of major bacterial genera.
Network connected with three beneficial bacterial genera that increased in the treatment group (genera Anaerostipes, Bifidobacterium 388775 and Fusicatenibacter).

## Slide 2
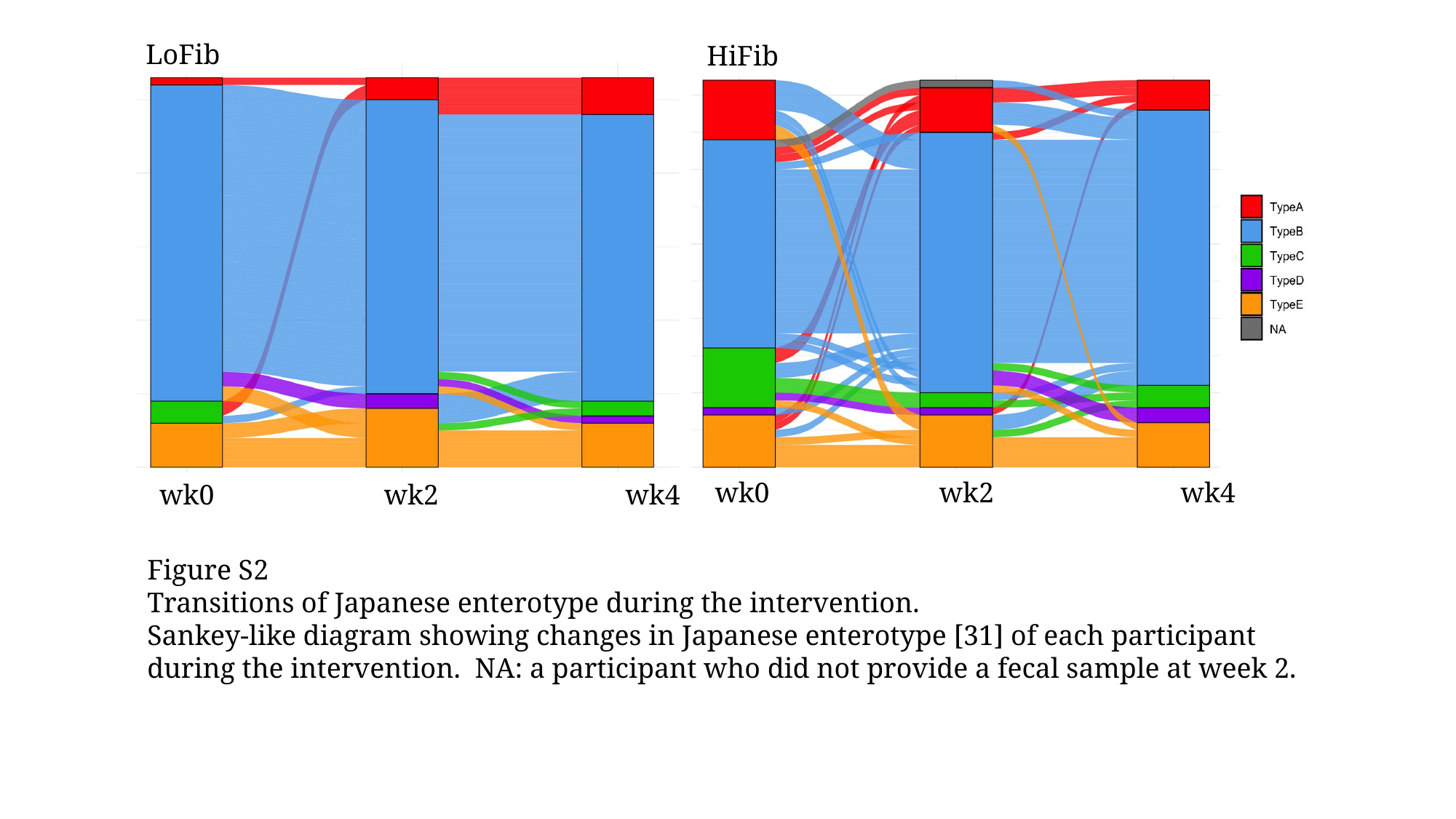

LoFib
 HiFib
wk0 wk2　　　 　 wk4
wk0 wk2　　　 　 wk4
Figure S2
Transitions of Japanese enterotype during the intervention.
Sankey-like diagram showing changes in Japanese enterotype [31] of each participant during the intervention. NA: a participant who did not provide a fecal sample at week 2.

## Slide 3
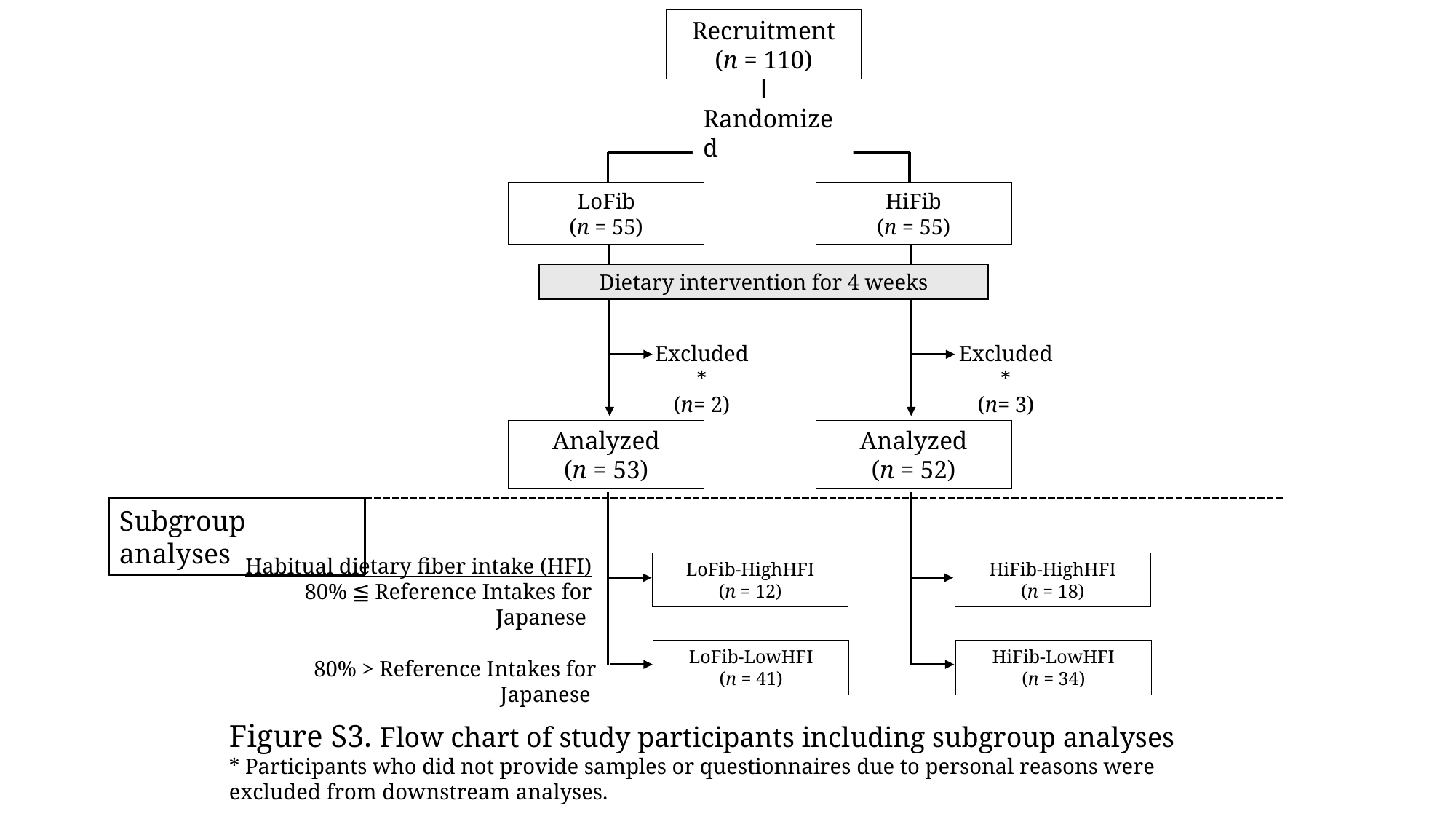

Recruitment
(n = 110)
Randomized
LoFib
(n = 55)
HiFib
(n = 55)
Dietary intervention for 4 weeks
Excluded*
(n= 2)
Excluded*
(n= 3)
Analyzed
(n = 53)
Analyzed
(n = 52)
Subgroup analyses
Habitual dietary fiber intake (HFI)
80% ≦ Reference Intakes for Japanese
LoFib-HighHFI
(n = 12)
HiFib-HighHFI
(n = 18)
LoFib-LowHFI
(n = 41)
HiFib-LowHFI
(n = 34)
80% > Reference Intakes for Japanese
Figure S3. Flow chart of study participants including subgroup analyses
* Participants who did not provide samples or questionnaires due to personal reasons were excluded from downstream analyses.

## Slide 4
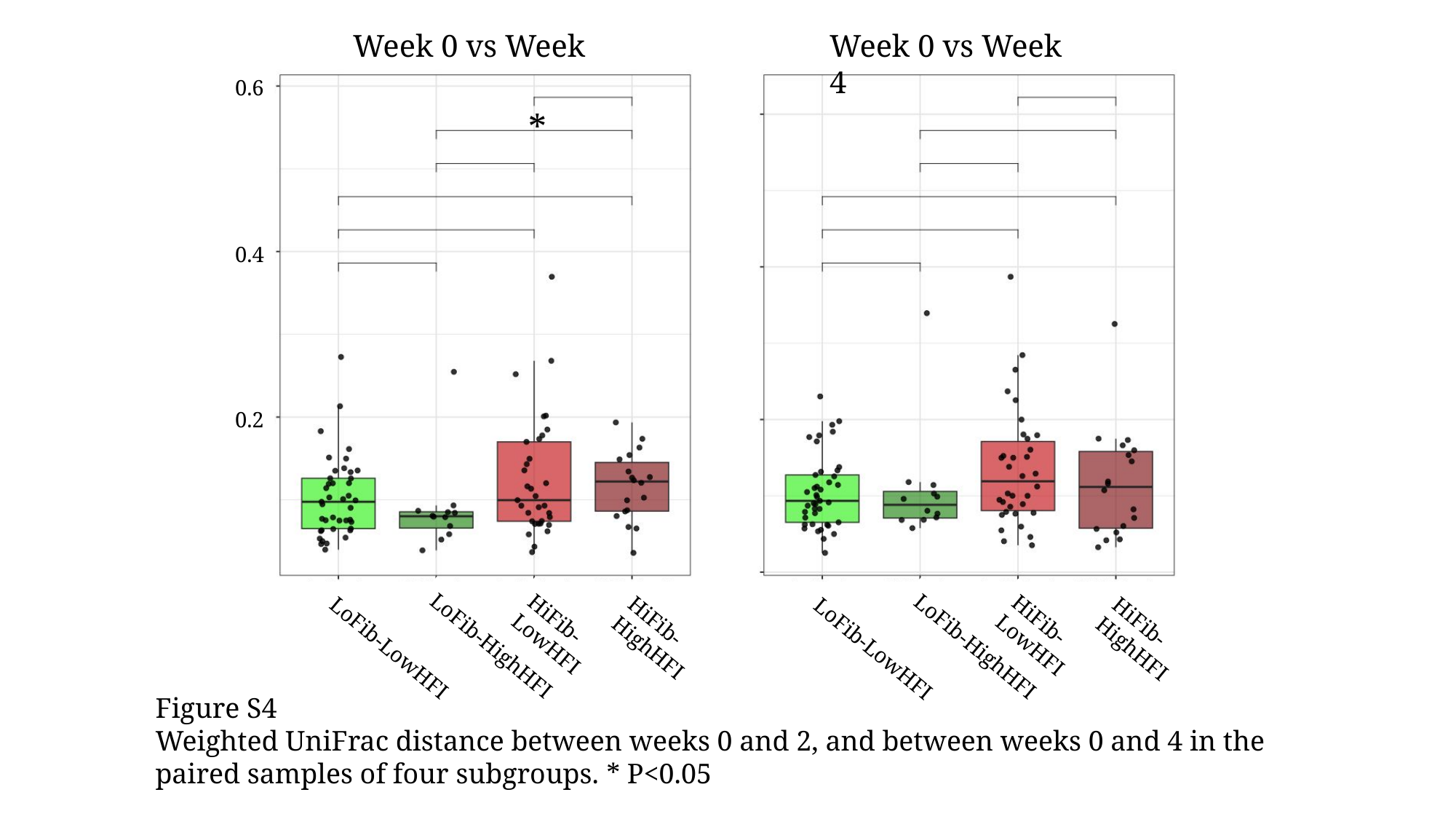

Week 0 vs Week 2
Week 0 vs Week 4
0.6
*
0.4
0.2
HiFib-LowHFI
HiFib-LowHFI
HiFib-HighHFI
LoFib-HighHFI
HiFib-HighHFI
LoFib-HighHFI
LoFib-LowHFI
LoFib-LowHFI
Figure S4
Weighted UniFrac distance between weeks 0 and 2, and between weeks 0 and 4 in the paired samples of four subgroups. * P<0.05

## Slide 5
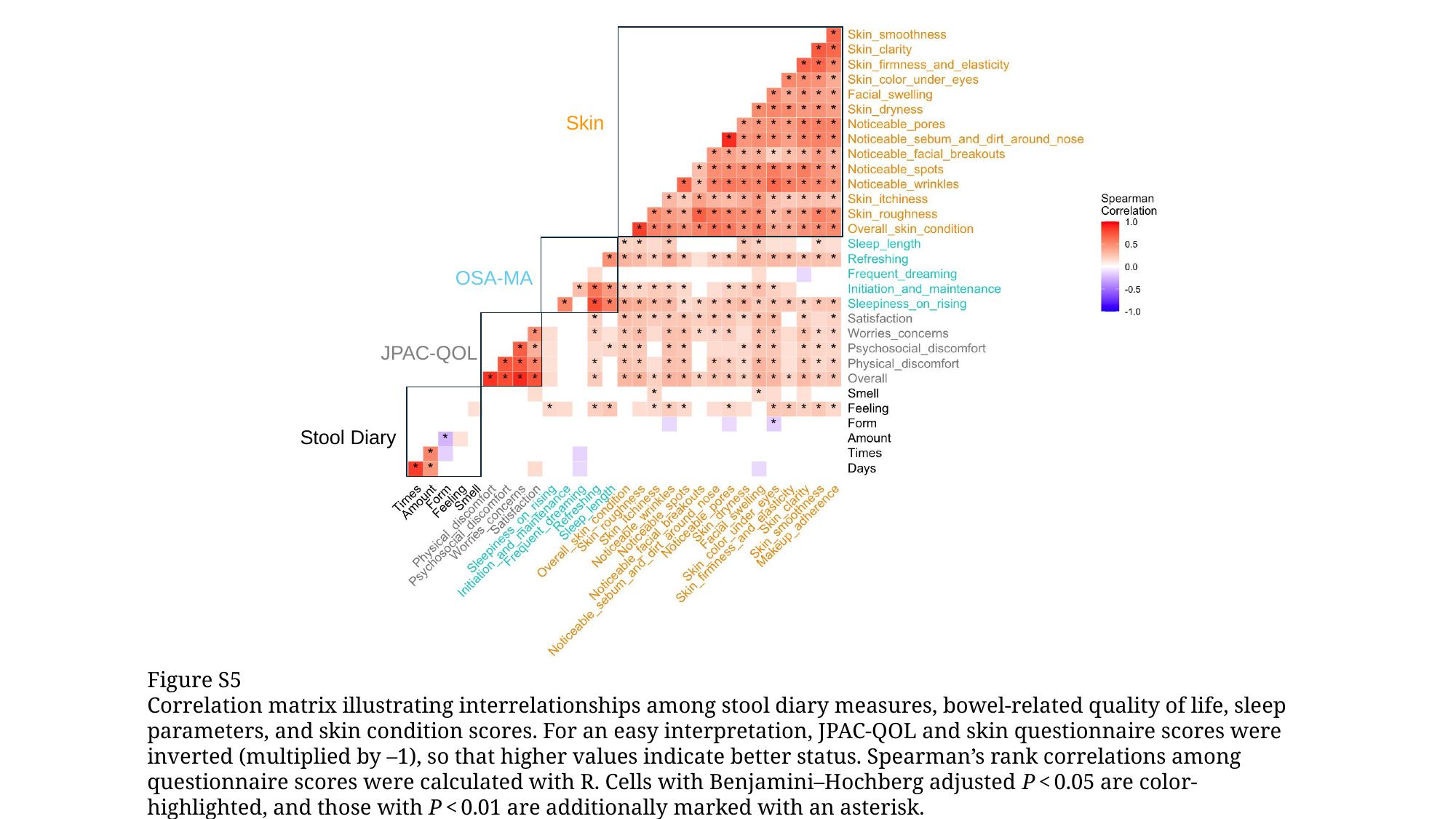

Skin
OSA-MA
JPAC-QOL
Stool Diary
Figure S5
Correlation matrix illustrating interrelationships among stool diary measures, bowel-related quality of life, sleep parameters, and skin condition scores. For an easy interpretation, JPAC-QOL and skin questionnaire scores were inverted (multiplied by –1), so that higher values indicate better status. Spearman’s rank correlations among questionnaire scores were calculated with R. Cells with Benjamini–Hochberg adjusted P < 0.05 are color-highlighted, and those with P < 0.01 are additionally marked with an asterisk.
