# Supplementary material for: Effects of Dietary Fiber Supplementation on Gut Microbiota and Bowel Function in Healthy Adults: A Randomized Controlled Trial
Source: Microorganisms. 2025 Sep 5;13(9):2068. doi: 10.3390/microorganisms13092068 (PMC12471990; doi:10.3390/microorganisms13092068)
Supplement: Supplementary file 1 [file microorganisms-13-02068-s001.zip › Text S1.pdf]

Supplementary Text S1

Method and Results of subgroup analysis focusing on habitual (baseline) dietary fiber intake

Method

Participants were stratified according to their habitual dietary fiber intake assessed by the short-FFQ. An intake less than 80% of the recommended level, as defined by the Dietary Reference Intakes for Japanese (2025 edition) [38], was considered low habitual fiber intake (LowHFI) and an intake above 80% was considered high habitual fiber intake (HighHFI). Combining this stratification with the randomized allocation (LoFib vs HiFib), participants were classified into four subgroups (LoFib-LowHFI, LoFib-HighHFI, HiFib-LowHFI, HiFib-HighHFI).

Results

The Low-HFI subgroup included 41 participants in the LoFib group and 34 in the HiFib group, whereas the High-HFI subgroup included 12 and 18 participants, respectively. Thus, the four subgroups consisted of: LoFib-LowHFI (n=41), HiFib-LowHFI (n=34), LoFib-HighHFI (n=12), and HiFib-HighHFI (n=18) (Figure S3). The mean habitual dietary fiber intake was 13.2 g and 16.1 g in the Low-HFI and High-HFI subgroups, respectively.

Effect of the intervention on questionnaires (Table S3)

In both LoFib-LowHFI and HiFib-LowHFI groups, an improvement in the information in the bowel-related questionnaires, namely stool diary and JAPC-QOL, were observed.

In contrast, in most questionnaires, especially JPAC-QOL, the information showed a greater improvement in the HiFib-HighHFI group than in the LoFib-HighHFI group.

Some information in the skin-related questionnaires also showed an improvement from weeks 0 to 2, and/or from weeks 0 to 4, but no obvious trends of the intervention or the baseline dietary fiber intake were found.

Sleep length improved only in the HiFib-HighHFI group.

Effect of the intervention on fecal organic acids (Table S3)

An increase in the concentrations of organic acids was observed in the HiFib-LoHFI and HiFib-HighHFI groups at week 2, but it was more obvious in the HiFib-HighHFI group. Only the concentrations of branched fatty acids, iso-butyrate and iso-valerate, increased in the

HiFib-LoHFI group. However, in the case of the HiFib-HighHFI group, in addition to those of branched fatty acids, the concentrations of propionate, n-butyrate and total SCFA (the sum of acetate, propionate, n-butyrate, n-valerate, iso-butyrate and iso-valerate) also increased. In the HiFib-HighHFI group, total SCFA also increased at week 4.

Effect of the intervention on the fecal microbiota

The indices for alpha diversity (Chao1 and Shannon) did not change during the intervention regardless of the treatment group or the baseline dietary fiber intake (data not shown).

Beta-diversity, namely the distance based on weighted UniFrac metrics between weeks 0 and 2, and between weeks 0 and 4 in paired samples was higher in the HiFib group than in the LoFib group regardless of habitual fiber intake, but the difference was significant only between LoFib-HighHFI and HiFib-HighHFI groups (Figure S4). Regardless of subgroups, the distance based on unweighted UniFrac did not differ both between weeks 0 and 2, and between weeks 0 vs 4 (data not shown).

Regarding the taxonomy at genus level, the relative abundances of 16, 3, 12 and 9 genera changed during the intervention in the LoFib-LowHFI, LoFib-HighHFI, HiFib-LowHFI and HiFib-HighHFI, respectively (Table S4). For example, the relative abundance of genus *Anaerostipes*, a butyrate-producing bacterium, decreased in the LoFib-LowHFI group but increased in the HiFib-LowHFI group. And, genus *Pseudobutyricicoccus* increased in the LoFib-HighHFI group at week 4, whereas genus *Coprobacter* increased in the HiFib-HighHFI group both at weeks 2 and 4.

Interestingly, the abundance of genus *Bifidobacterium* 388775 increased in the LoFib-LowHFI but not in HiFib-LowHFI group. On the other hands, it increased in the HiFib-HighHFI group but not in LowFib-HighHFI.
